# Comparison of Duloxetine Supplemented With Pregabalin and Amitriptyline Supplemented With Pregabalin for the Treatment of Postherpetic Neuralgia: A Double‐Blind, Randomized Crossover Trial

**DOI:** 10.1111/cns.70460

**Published:** 2025-05-26

**Authors:** Yue Wang, Chenchen Wu, Yanan Liu, Youpan Lou, Cheng Chen, Qianqian Chen, Xihan Li, Chao Ma, Jing Li, Ying Huang

**Affiliations:** ^1^ Department of Pain Nanjing Drum Tower Hospital Clinical College of Nanjing University of Chinese Medicine Nanjing China; ^2^ Department of Gastroenterology Nanjing Drum Tower Hospital Clinical College of Nanjing University of Chinese Medicine Nanjing China; ^3^ Department of Pain Nanjing Drum Tower Hospital, Affiliated Hospital of Medical School, Nanjing University Nanjing China

**Keywords:** amitriptyline, duloxetine, postherpetic neuralgia, pregabalin

## Abstract

**Background:**

Postherpetic neuralgia (PHN) is the most common chronic complication of herpes zoster (HZ), and current treatment regimens often fail to effectively control the pain.

**Aim:**

The purpose of this study is to compare the efficacy and tolerability of duloxetine combined with pregabalin and amitriptyline combined with pregabalin in the treatment of PHN, as well as their impact on sleep and quality of life in patients with PHN.

**Methods:**

This is a double‐blind, randomized, crossover trial involving PHN patients. About 220 participants were randomly assigned (1:1) to either the duloxetine combined with pregabalin or the amitriptyline combined with pregabalin. The patient takes duloxetine or amitriptyline orally before bedtime for 6 weeks each time. Perform a 2‐week single‐blind placebo washout between the two treatments, and a 2‐week single‐blind placebo washout at the end of the treatment period. In the last week of the treatment cycle, evaluate the 7‐day average daily pain, Pittsburgh Sleep Quality Index, 17‐item Hamilton Depression Rating Scale, 36‐Item Short Form Health Survey, and record the occurrence of major adverse events. A median reduction of 50%, 25%–50%, and 25% in pain score is considered good, moderate, and mild improvement, respectively.

**Results:**

Both treatment methods can significantly improve the baseline pain value (*p* < 0.001 for both). The combination of duloxetine and pregabalin resulted in good, moderate, and mild pain relief of 52%, 24%, and 7%, respectively. The combination of amitriptyline and pregabalin resulted in good, moderate, and mild pain relief of 48%, 21%, and 9%, respectively. There was no significant difference in the measurement of various results between the two groups. In the reported adverse events, patients in the amitriptyline group had significantly more dry mouth compared to the duloxetine group (26% vs. 11%; *p* = 0.008).

**Conclusions:**

Duloxetine and amitriptyline have similar analgesic effects, and the difference is not statistically significant. The combination of pregabalin has good tolerability and better pain relief in PHN patients, thus providing clinically relevant benefits.

**Trial Registration:** Chinese Clinical Trial Registry: ChiCTR2100054831

## Graphical abstract

This is a double‐blind, randomized, crossover trial involving PHN patients. The purpose of this study is to compare the efficacy and tolerability of duloxetine combined with pregabalin and amitriptyline combined with pregabalin in the treatment of PHN, as well as their impact on sleep and quality of life in patients with PHN.

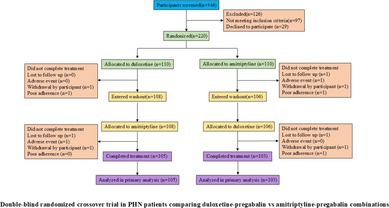

## 1 Introduction

Herpes zoster (HZ) is an infectious disease affecting the nerves and skin, caused by the varicella‐zoster virus, which is characterized by blisters clustered along the unilateral peripheral nerve, often accompanied by neurological symptoms [1]. Postherpetic neuralgia (PHN) is usually defined as skin pain that lasts for at least 90 days after the acute HZ rash outbreak [2]. It is the most common complication of HZ, and the pain affects the patient's general activity, sleep, and emotions [3]. Patients with severe pain are more likely to experience anxiety and depression symptoms, which can last for months or even years, seriously affecting the patient's quality of life [4].

At present, first‐line treatment drugs based on guidelines include tricyclic antidepressants, calcium channel modulators (pregabalin, gabapentin), lidocaine patches, etc. [5]. The most commonly used tricyclic antidepressants are amitriptyline, which can inhibit the reuptake of serotonin and norepinephrine, as well as have sedative and anticholinergic effects [6]. However, due to its ability to simultaneously block histamine H1 receptors, noradrenergic A1 receptors, and cholinergic M1 receptors, amitriptyline may cause anticholinergic side effects (e.g., dry mouth, constipation) that limit tolerability [7]. Duloxetine, a SNRI antidepressant, is a second‐line treatment recommended in the guidelines for the treatment of PHN [8]. It can selectively inhibit the reuptake of serotonin and norepinephrine, with a potentially favorable safety profile [9]. This study aims to compare the clinical efficacy and tolerability of duloxetine hydrochloride combined with pregabalin and amitriptyline combined with pregabalin in PHN, in order to provide clinically relevant benefits for PHN patients.

## 2 Methods

### 2.1 Study Design and Participants

This clinical trial has obtained ethical approval from the Ethics Committee of Nanjing Drum Tower Hospital (2021‐393‐02). This trial was registered with the Chinese Clinical Trial Registry (ChiCTR2100054831) prior to patient enrollment. All methods and results were reported in accordance with the CONSORT guidelines. We obtained written informed consent from all participants prior to beginning the research process.

Our study recruited patients between the ages of 18 and 80 diagnosed with PHN. Participants who meet the criteria must have an average pain intensity score greater than 4 measured on a numerical rating scale (NRS, 0 = no pain, 10 = most severe pain). Those who have previously been exposed to PHN drugs, regardless of dosage and duration, are considered for inclusion after a 2‐week placebo washout period.

Exclusion criteria include refusal to participate in the study or provide written informed consent, liver, kidney, or immune dysfunction, mental instability, cognitive impairment, neurological disorders caused by other reasons, uncontrolled hypertension, recent heart or brain ischemia events, pregnancy or breastfeeding, allergies to study drugs, malignant tumors, drug or alcohol abuse. In addition, participants who participated in another clinical trial 3 months before entering the study were also excluded.

### 2.2 Randomization and Masking

Participants who meet the eligibility criteria were randomly assigned to either the duloxetine plus pregabalin group or the amitriptyline plus pregabalin group (1:1). Randomization was conducted by an independent research assistant using a network‐based central randomization system, who was not involved in any other aspects of the study. This is a double‐blind study. To maintain blinding, duloxetine and amitriptyline tablets were encapsulated in identical opaque gelatin capsules by an independent compounding pharmacy. Pregabalin was administered as commercially available capsules. Participants and investigators were unaware of treatment assignments throughout the trial. The evaluation results and analysis data were also concealed from the treatment drugs.

### 2.3 Procedures

This is a randomized, double‐blind, crossover clinical trial. All participants undergo a 2‐week washout period and stop taking their current painkillers for treating PHN. A washout period of 2 weeks was implemented between treatment phases to mitigate potential carryover effects. This duration was determined based on the pharmacokinetic profile of amitriptyline, which has an elimination half‐life of approximately 31–46 h. Five half‐lives (5 × *t*_1/2_) were calculated to ensure > 97% drug clearance before initiating the second intervention.

Participants were randomly assigned to either the duloxetine combined with pregabalin group or the amitriptyline combined with pregabalin group (1:1). Duloxetine is provided by Lilly Del Caribe Inc., amitriptyline is provided by Changzhou Siyao Pharmaceuticals Co. Ltd., and pregabalin is provided by Pfizer Pharmaceuticals Co. Ltd. The treatment starts with a low dose of duloxetine 30 mg, once a day, before bedtime; amitriptyline 25 mg once a day before bedtime; pregabalin 150 mg, twice daily, increasing the dose of duloxetine or amitriptyline every 2 weeks based on pain assessment. Duloxetine has a maximum daily dose of 90 mg, while amitriptyline has a maximum daily dose of 75 mg. For participants who experience adverse reactions, the dosage will be adjusted to the final tolerance level. As a rescue medication for pain control, patients are allowed to take 1000 mg of acetaminophen for pain treatment, with a maximum daily dose of 4000 mg.

After 6 weeks of drug treatment, each group of participants underwent a 2‐week washout period, then received a 6‐week cross treatment plan. At the end of the 14th week of the study, patients entered a 2‐week placebo treatment period and were evaluated. During the entire follow‐up period, the use of intrathecal, epidural, or peripheral nerve block therapy is prohibited.

### 2.4 Study Outcomes

#### 2.4.1 Primary Outcome

The primary outcome is NRS, which is recorded using a pain rating method. A ruler with a scale and a length of 10 cm is used, and each scale is marked with 11 numbers from 0 to 10 in order. 0 = no pain, 10 = severe pain, and evaluations are conducted every 2 weeks from the start of treatment.

#### 2.4.2 Secondary Outcomes

Pain relief is measured by overall patient efficacy evaluation, with overall improvement and adverse events as secondary outcomes. At weeks 4, 8, 12, and 16, 7‐day average daily pain, Pittsburgh Sleep Quality Index (PSQI), 17‐item Hamilton Depression Rating Scale (HAMD‐17), and 36‐Item Short Form Health Survey (SF‐36) are evaluated [10, 11, 12]. The PSQI self‐assessment questionnaire was used to measure sleep quality, with scores ranging from 0 to 21. A higher score indicates more sleep disorders, with a PSQI score > 5 defined as poor sleep quality. The HAMD‐17 Depression Scale includes 17 items, among which mild depression: HAMD‐17 item scores > 7 and ≤ 17 points; moderate depression: HAMD‐17 score > 17 and ≤ 24 points; severe depression: HAMD‐17 score > 24 points. The factor components include anxiety, somatization, weight changes, cognitive impairment, blockade, sleep disorders, etc. SF‐36 is used to evaluate quality of life (QOL), including 8 assessment items: physical functioning (PF), role physical (RP), bodily pain (BP), general health (GH), vitality (VT), social functioning (SF), role emotional (RE), and mental health (MH). Each item is scored 100 points, and the higher the score, the better the quality of life.

A median reduction of 50%, 25%–50%, and 25% in pain score is considered good, moderate, and mild improvement, respectively. All parameters of population characteristics are recorded. The occurrence, severity, and duration of adverse reactions are also recorded in the patient's diary.

### 2.5 Statistical Analysis

The calculation of sample size is based on observing the improvement rate of PHN pain score and referring to previous clinical trials of duloxetine compared to amitriptyline. For the 2 × 2 crossover design, using a unilateral non‐inferiority test, at a non‐inferiority threshold of 15%, *β* = 90%, significance level of 0.05, and a SD of 0.7 for paired differences, the sample size for each group is 94, for a total of 188 participants. We plan to include 220 participants to compensate for 15% of the follow‐up loss.

This study conducted a baseline comparability analysis and strictly followed the pre‐established statistical analysis plan. The data from the per‐protocol set were used for analysis, and missing data were deleted. The statistical analysis of the data was conducted using SPSS 28.0 software, and normality of data distribution was assessed using the Kolmogorov–Smirnov test. Data with a normal distribution were expressed as mean ± standard deviation (^−^ *x* ± SD) and analyzed using parametric tests (Student's *t*‐test, repeated measures ANOVA), while non‐normally distributed data were described using interquartile range (IQRs) and analyzed using non‐parametric equivalents (Mann–Whitney *U* test, Wilcoxon signed‐rank test). The count data are expressed as a percentage, and the comparison between the two groups is conducted using the chi‐squared test. Categorical variables are represented as numbers (%) and compared using chi‐squared test or Fisher's exact test. The comparison of quantitative data at different time points was conducted using repeated measures analysis of variance, with *p* < 0.05 indicating statistically significant differences.

To address potential confounding factors in the crossover design, a post hoc sensitivity analysis was performed using a linear mixed‐effects model (LMM). The model included fixed effects for treatment group (duloxetine vs. amitriptyline), study phase, treatment period, age (*Z*‐score standardized), and baseline pain severity (standardized). Sensitivity analyses confirmed the robustness of the results.

## 3 Results

### 3.1 Patients Characteristics

**FIGURE 1:**
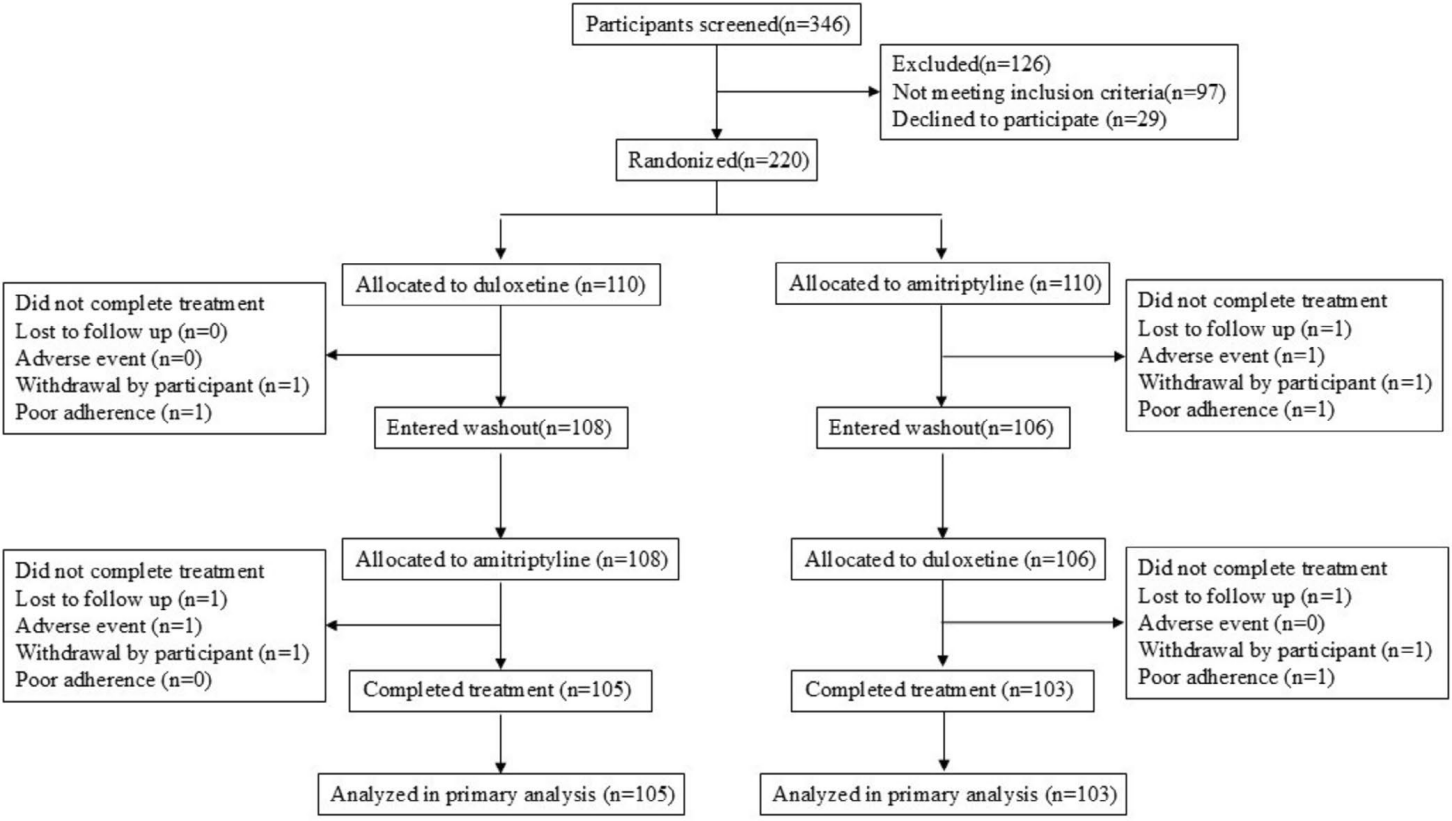
CONSORT statement.

From March 17, 2022 to August 11, 2023, a total of 346 patients were pre screened and evaluated. Ninety seven patients did not meet the inclusion criteria, and 29 patients refused to participate. Two hundred and twenty patients were randomized into two groups. One hundred and ten participants were randomly assigned to the duloxetine combined with pregabalin group, and 110 participants were assigned to the amitriptyline combined with pregabalin group (Figure 1). Among the randomly assigned patients, 12 terminated the study, with 3 losing follow‐up, 2 due to adverse events, 4 due to personal reasons, and 3 due to poor adherence. Finally, 105 patients (95.5%) in the duloxetine combined with pregabalin group and 103 patients (93.6%) in the amitriptyline combined with pregabalin group completed the 16‐week study period. The demographic and clinical characteristics of the two groups of patients are similar (Table 1).

**TABLE 1:** Basic demography of randomized participants.

Characteristics	Duloxetine group (*n* = 110)	Amitriptyline group (*n* = 110)
Sex, *N* (%)		
Male	58 (52.7)	51 (46.4)
Female	52 (47.3)	59 (53.6)
Age, mean (SD), years	69.3 (7.8)	68.5 (8.6)
PHN location, *N* (%)		
Thorax	49 (44.5)	45 (40.9)
Abdomen	28 (25.5)	30 (27.3)
Extremities	21 (19.1)	19 (17.3)
Others	12 (10.9)	16 (14.5)
Duration of neuropathic pain, mean (SD), months	11.3 (4.6)	10.9 (5.2)
NRS pain, mean (SD)	5.4 (0.8)	5.5 (0.9)

*Note:* Data are mean (SD) or *n* (%).

Abbreviations: NRS, numerical rating scale; PHN, postherpetic neuralgia.

### 3.2 Efficacy

#### 3.2.1 Primary Outcome

**FIGURE 2:**
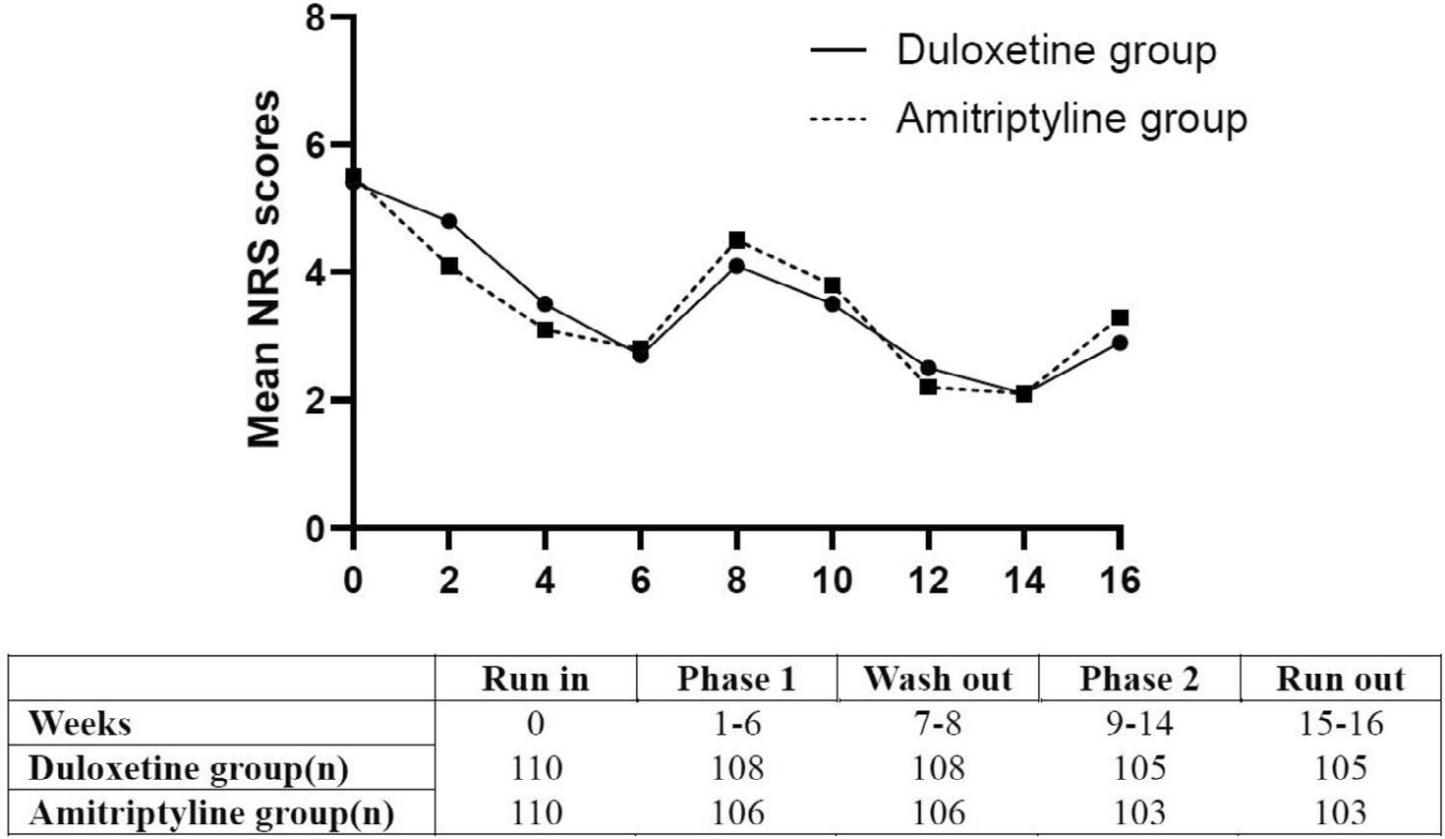
Median NRS pain scores of participants. NRS, numerical rating scale.

Figure 2 shows the primary outcome of subjective pain. During the 16‐week study period, duloxetine combined with pregabalin and amitriptyline combined with pregabalin both reduced NRS levels (*p* < 0.001 for both), but there was no significant difference between the two groups, and neither group showed better performance than the other.

#### 3.2.2 Secondary Outcomes

The PSQI and HAMD‐17 scores of patients in the duloxetine combined with pregabalin group and amitriptyline combined with pregabalin group decreased significantly after treatment compared to before treatment (all *p* < 0.001, Table 2). However, there was no statistically significant difference in PSQI and HAMD‐17 scores between the two groups of patients after treatment (*p* = 0.972 and *p* = 0.748).

**TABLE 2:** Patients' general well‐being through subjective assessments and daytime performance measures.

	Duloxetine group (*n* = 105)	Amitriptyline group (*n* = 103)	*p*
PSQI scores, mean (SD)			
Baseline	15.5 (4.9)	15.2 (4.8)	0.656
At week 4	8.5 (3.2)	9.2 (2.2)	0.068
At week 8	9.6 (2.6)	10.3 (2.8)	0.063
At week 12	7.7 (2.2)	8.2 (2.0)	0.088
At week 16	7.9 (2.4)	8.5 (2.1)	0.057
HAMD‐17 scores, mean (SD)			
Baseline	14.2 (3.9)	14.1 (4.2)	0.859
At week 4	9.7 (3.0)	10.4 (3.2)	0.105
At week 8	10.1 (3.1)	10.8 (3.2)	0.111
At week 12	7.8 (2.4)	8.1 (3.0)	0.426
At week 16	8.5 (2.7)	8.7 (3.1)	0.620
SF‐36 physical summary			
Baseline	20.4 (6.3)	21.2 (6.7)	0.376
At week 4	26.1 (9.3)	25.6 (9.0)	0.694
At week 8	24.1 (7.1)	23.6 (7.3)	0.617
At week 12	28.1 (8.5)	27.4 (7.9)	0.539
At week 16	25.8 (7.7)	26.0 (8.0)	0.854
SF‐36 mental summary			
Baseline	35.6 (11.2)	34.4 (13.0)	0.476
At week 4	41.7 (13.4)	39.1 (12.8)	0.154
At week 8	40.2 (11.6)	38.5 (12.1)	0.302
At week 12	44.5 (13.2)	44.0 (13.4)	0.787
At week 16	41.9 (12.4)	41.3 (13.0)	0.734

The SF‐36 scores of patients in the duloxetine combined with pregabalin group and amitriptyline combined with pregabalin group increased after treatment compared to before treatment, and the difference was statistically significant (all *p* < 0.001, Table 2). However, there was no statistically significant difference in SF‐36 physical summary and SF‐36 mental summary scores between the two groups of patients after treatment (*p* = 0.947 and *p* = 0.713).

The combination therapy of duloxetine and pregabalin can alleviate 52%, 24%, and 7% of good, moderate, and mild pain, respectively. The combination therapy of amitriptyline and pregabalin can alleviate 48%, 21%, and 9% of good, moderate, and mild pain, respectively. There was no significant difference between the two groups (*p* = 0.351).

#### 3.2.3 Adverse Events

This study reported adverse events such as somnolence, insomnia, dizziness, fatigue, dry mouth, nausea, diarrhea, etc. [13]. Out of 105 patients in the combination of duloxetine and pregabalin group, 92 events were found, while in the combination of amitriptyline and pregabalin group, 105 events were found (Table 3). Overall, the most common adverse events in the combination of duloxetine and pregabalin group were somnolence (18 events [17%]), fatigue (13 events [12%]), and dry mouth (12 events [11%]). The most common adverse reactions in the combination of amitriptyline and pregabalin group were dry mouth (27 events [26%]), somnolence (15 events [15%]), and fatigue (14 events [14%]). Two patients withdrew from the study due to intolerable adverse events. Among all adverse events, dry mouth was more common in the combination of amitriptyline and pregabalin group (*p* = 0.008).

**TABLE 3:** Adverse events observed.

Adverse events	Duloxetine (*n* = 105)	Amitriptyline (*n* = 103)	*p*
Somnolence	18 (17%)	15 (15%)	0.705
Dry mouth	12 (11%)	27 (26%)	0.008
Insomnia	8 (8%)	9 (9%)	0.805
Dizziness	7 (7%)	6 (6%)	1.000
Fatigue	13 (12%)	14 (14%)	0.839
Nausea	5 (5%)	4 (4%)	1.000
Constipation	9 (9%)	7 (7%)	0.796
Diarrhea	10 (10%)	12 (12%)	0.658
Pain abdomen	2 (2%)	1 (1%)	1.000
Itching	1 (1%)	2 (2%)	0.620
Excessive sweating	7 (7%)	8 (8%)	0.795

*Note:* Data are *n* (%).

### 3.3 Mixed‐Effects Model Adjusted for Covariates

After adjusting for age and baseline pain severity using the mixed‐effects model, the estimated treatment difference between duloxetine and amitriptyline remained not significant (*p* = 0.129), consistent with the primary results (*p* = 0.143). Age showed no significant association with treatment outcome (*p* = 0.069), whereas higher baseline pain severity was associated with greater endpoint pain scores (*p* = 0.034).

## 4 Discussion

At present, the first‐line treatment drugs for PHN based on guidelines include tricyclic antidepressants, calcium channel modulators, and lidocaine patches [14]. The most commonly used tricyclic antidepressant is amitriptyline, which inhibits PHN, reduces anxiety symptoms, and improves sleep by increasing serotonin and noradrenaline levels between synapses, blocking sodium ion conduction on neurons, stimulating endogenous adenosine receptors, and activating α2 receptors [15]. Due to its effect on cholinergic receptors, long‐term use of amitriptyline can cause various adverse reactions, such as dry mouth, constipation, and drowsiness, which often lead to poor patient tolerance [16].

Duloxetine is a second‐line drug recommended for the treatment of PHN and the only SNRI antidepressant recommended in the guidelines [8, 9]. It can enhance neurotransmitter transmission by inhibiting the reuptake of serotonin and norepinephrine by central neurons in the brain and spinal cord, thereby enhancing the function of the descending inhibitory pathway and restoring the balance of the descending inhibitory and facilitation systems [17]. At the same time, it reduces the transmission of spinal pain signals, enhances the body's tolerance to pain, increases the body's pain threshold, and improves sleep quality to achieve the goal of relieving pain [18]. Duloxetine's absorption in the body is not affected by drugs and food and can be fully absorbed in the gastrointestinal tract [19]. It has strong lipophilicity and will not be destroyed by various enzymes in gastric and intestinal fluids [20]. Therefore, it is absorbed quickly in the body and can be quickly and widely distributed in various tissues. Chunmei et al. reported that duloxetine can reduce the incidence rate of post herpetic neuralgia in patients with HZ and proposed to use duloxetine to prevent the occurrence of post herpetic neuralgia [20].

Pregabalin is a drug that binds to a specific ligand of the voltage‐gated calcium channel α2δ helper subunit [21]. It can alleviate PHN, improve sleep and emotional disorders, and is recommended by experts as a first‐line treatment for PHN [22]. However, patients using pregabalin still experience explosive pain, and poor sleep and inadequate pain control are common during the initial treatment stage. In recent years, there have been many clinical studies on the combination of pregabalin and other drugs for treatment [23]. Dworkin et al. believe that pregabalin is safe for treating PHN, effective in relieving pain and sleep disturbances, and has a greater overall improvement compared to placebo treatment [24]. Manlio Barbarisi et al. found that the combination therapy of pregabalin and TENS has a good therapeutic effect on improving pain in patients with PHN [25].

This study used duloxetine hydrochloride combined with pregabalin to treat PHN and evaluated whether the combination therapy could improve pain and quality of life in PHN patients. At the same time, we used amitriptyline combined with pregabalin as a control in PHN patients. The expected results of this study have a certain impact on the treatment guidelines for PHN patients. Our research results indicate that the combination of duloxetine hydrochloride and pregabalin in the treatment of PHN patients significantly reduces pain scores compared to before treatment and improves sleep quality and depression status. Its pain relief, sleep improvement, and depression status are comparable to those of the amitriptyline group, indicating that the combination of duloxetine hydrochloride and pregabalin can effectively alleviate pain, improve sleep quality, and depression status in PHN patients. At the same time, the incidence of adverse reactions to duloxetine hydrochloride combined with pregabalin is low, especially in the dry mouth group, which is significantly lower than that of the amitriptyline group. Duloxetine's pharmacoeconomic advantage emerges from its lower TEAE(treatment emergent adverse event) rates (12% vs. 27% dry mouth), reducing ancillary treatment costs. QALY analysis showed 0.12 incremental gain versus amitriptyline (95% CI 0.05–0.19). For patients who cannot tolerate the side effects of amitriptyline, duloxetine hydrochloride combined with pregabalin can be used to treat PHN, but more research is needed on its duration and dosage. There were no significant safety issues in our trial, and the dose of duloxetine was well tolerated, similar to other clinical trials of duloxetine.

However, several limitations of this study should be addressed. First, due to the lack of placebo, the study was partially limited. Second, the study period of this experiment is limited to 16 weeks, and longer follow‐up studies should be conducted in the future.

## 5 Conclusion

In summary, the combination of duloxetine hydrochloride and pregabalin is effective in treating PHN, helping PHN patients relieve pain, improve sleep quality, and alleviate depression. It has few adverse reactions and high safety, and is worthy of clinical promotion and application.

## Author Contributions

Y.H. and C.M. were involved in the development and design of the study concept. Y.W. and C.W. contributed to the initial manuscript writing. Y.L. and Y.L. were involved in intervention and assessment. J.L., C.C., Q.C., and X.L. were involved in data acquisition and analysis. All authors revised and agreed to the final version of this article.

## Conflicts of Interest

The authors declare no conflicts of interest.

## Data Availability

The data that support the findings of this study are available from the corresponding author upon reasonable request.
